# Two-dimensional characterization of three-dimensional magnetic bubbles in Fe_3_Sn_2_ nanostructures

**DOI:** 10.1093/nsr/nwaa200

**Published:** 2020-08-28

**Authors:** Jin Tang, Yaodong Wu, Lingyao Kong, Weiwei Wang, Yutao Chen, Yihao Wang, Y Soh, Yimin Xiong, Mingliang Tian, Haifeng Du

**Affiliations:** Anhui Province Key Laboratory of Condensed Matter Physics at Extreme Conditions, High Magnetic Field Laboratory of the Chinese Academy of Sciences, and University of Science and Technology of China, Hefei 230031, China; Anhui Province Key Laboratory of Condensed Matter Physics at Extreme Conditions, High Magnetic Field Laboratory of the Chinese Academy of Sciences, and University of Science and Technology of China, Hefei 230031, China; Universities Joint Key Laboratory of Photoelectric Detection Science and Technology in Anhui Province, Hefei Normal University, Hefei 230601, China; School of Physics and Materials Science, Anhui University, Hefei 230601, China; Institute of Physical Science and Information Technology, Anhui University, Hefei 230601, China; Anhui Province Key Laboratory of Condensed Matter Physics at Extreme Conditions, High Magnetic Field Laboratory of the Chinese Academy of Sciences, and University of Science and Technology of China, Hefei 230031, China; Anhui Province Key Laboratory of Condensed Matter Physics at Extreme Conditions, High Magnetic Field Laboratory of the Chinese Academy of Sciences, and University of Science and Technology of China, Hefei 230031, China; Paul Scherrer Institute, 5232 Villigen, Switzerland; Anhui Province Key Laboratory of Condensed Matter Physics at Extreme Conditions, High Magnetic Field Laboratory of the Chinese Academy of Sciences, and University of Science and Technology of China, Hefei 230031, China; Anhui Province Key Laboratory of Condensed Matter Physics at Extreme Conditions, High Magnetic Field Laboratory of the Chinese Academy of Sciences, and University of Science and Technology of China, Hefei 230031, China; School of Physics and Materials Science, Anhui University, Hefei 230601, China; Anhui Province Key Laboratory of Condensed Matter Physics at Extreme Conditions, High Magnetic Field Laboratory of the Chinese Academy of Sciences, and University of Science and Technology of China, Hefei 230031, China; Institute of Physical Science and Information Technology, Anhui University, Hefei 230601, China

**Keywords:** skyrmion, skyrmion bubbles, three-dimensional magnetic structures, differential phase contrast scanning transmission electron microscopy, micromagnetics

## Abstract

We report differential phase contrast scanning transmission electron microscopy (TEM) of nanoscale magnetic objects in Kagome ferromagnet Fe_3_Sn_2_ nanostructures. This technique can directly detect the deflection angle of a focused electron beam, thus allowing clear identification of the real magnetic structures of two magnetic objects including three-ring and complex arch-shaped vortices in Fe_3_Sn_2_ by Lorentz-TEM imaging. Numerical calculations based on real material-specific parameters well reproduced the experimental results, showing that the magnetic objects can be attributed to integral magnetizations of two types of complex three-dimensional (3D) magnetic bubbles with depth-modulated spin twisting. Magnetic configurations obtained using the high-resolution TEM are generally considered as two-dimensional (2D) magnetic objects previously. Our results imply the importance of the integral magnetizations of underestimated 3D magnetic structures in 2D TEM magnetic characterizations.

## Teaser

We demonstrate three-dimensional magnetic bubbles for the first time from the view of integral magnetizations, which clarify the physics behind complex multi-ring and arc-shaped vortices obtained from two-dimensional transmission electron microscopy magnetic imaging.

## INTRODUCTION

Magnetic skyrmions are topologically nontrivial nanometric spin whirls that are expected to be information carriers in future energy-efficient spintronic devices [1–19]. They were first found in non-centrosymmetric magnetic compounds, where chiral Dzyaloshinskii–Moriya interactions (DMIs) bend the magnetic moments [20–23]. The unique feature of magnetic skyrmions is their nontrivial topology defined by unit topological charge [24]. Unlike the chiral DMI-induced skyrmions, magnetic bubbles originate from the interplay of four types of interactions, including ferromagnetic exchange coupling, dipolar–dipolar interaction (DDI), uniaxial anisotropy and Zeeman energy. Competition among the first three interactions leads to stripe domains, which may change into a magnetic bubble when applying an external field. There are two types of magnetic bubbles according to the rotation sense of the cylinder domain wall (Fig. S1). One is a type-I magnetic bubble stabilized by a perpendicular magnetic field with a clockwise or anticlockwise closure cylinder domain wall contributing to a similar integer topological winding number as a skyrmion; type-I magnetic bubbles are thus renamed skyrmion bubbles [25–28]. The other one is a type-II magnetic bubble stabilized by a tilted magnetic field with magnetization in the partially reversed cylinder domain wall, with all domain wall magnetizations pointing toward the in-plane field component. However, such a domain wall in a type-II magnetic bubble contributes to a zero winding number and is topologically trivial [27]. The first wave of interest in magnetic bubbles occurred in the 1970s–1980s, motivated by experimental and theoretical studies of potential bubble memory [29,30]. The detection of skyrmion bubbles renewed the interest in magnetic bubbles in the last decade [25–28,31–35].

Although these two types of bubbles are well understood within the theoretical framework describing uniaxial ferromagnets, a recent study on a typical uniaxial ferromagnet Fe_3_Sn_2_ found new exotic spin whirls beyond conventional magnetic bubbles by Lorentz transmission electron microscopy (Lorentz-TEM) [25,28]. Two typical examples of such new spin whirls are three-ring and complex arch-shaped vortices characterized by a series of concentric circular stripe domains and one or multiple bound pairs of rotating magnetic whirls, respectively. Such magnetic structures were also observed in other uniaxial ferromagnets [26,31]. These objects are nanoscale size, which implies that they can be applied as information carriers in spintronic devices [17]. However, they are neither detected by other magnetic imaging methods nor in simulations conducted under realistic conditions. Moreover, a recent study demonstrated that the improper filter parameter in the transport of intensity equation (TIE) analysis of Lorentz-TEM imaging of type-II bubbles can lead to artificial biskyrmion structures [33].

Three-dimensional (3D) magnetic structures have become an active research topic because they are important in understanding novel experimental phenomena and potential applications [4,23,36–40]. It has been suggested that the chiral exchange interactions play important roles in tailoring 3D magnetic structures in synthetic antiferromagnets for potential 3D spintronic systems [39,40]. 3D magnetic skyrmions in *B*_20_ magnets induced by DMI have been proposed to understand the stability of zero-field target skyrmions and attractive interactions between skyrmions [4,23]. Magnetic skyrmion bubbles have also been predicted with depth-modulated spin twisting induced by DDI [41]. One typical characteristic of 3D magnetic skyrmion bubbles is that skyrmions near two surfaces have nearly contrary Néel twisting. This characteristic has been observed in magnetic multilayers by some surface-sensitive magnetic detection methods [36–38]. TEM is a real-space imaging of integral magnetic field over depth with ultrahigh spatial resolution. Magnetic configurations in thin nanostructures have been typically considered as quasi-two-dimensional (quasi-2D) magnetic objects using TEM [19,25,26,28,31]. However, one may clarify real 3D magnetic structures from the difference in integral magnetization over depth. This rule has been used to identify 3D chiral bobbers from integral phase shifts weaker than skyrmion tubes using TEM [3]. The depth-modulated 3D magnetic bubbles are also expected to show more complex integral magnetizations over the depth and are detected using 2D TEM magnetic imaging. The underestimated complex integral magnetizations of 3D magnetic bubbles may clarify the physics behind the complex three-ring and arch-shaped vortices in Fe_3_Sn_2_ through TEM, which is more readily considered as 2D magnetic configurations in thin nanostructures [25].

Here, we investigate the magnetic objects in an Fe_3_Sn_2_ nanodisk using differential phase contrast scanning transmission electron microscopy (DPC-STEM) combined with micromagnetic simulations. The observed magnetic objects are clarified as 2D integral magnetizations of complex 3D type-I and type-II bubbles with depth-modulated configurations. The characterization is considered further such that the origin of the artificial magnetic configurations detected in Lorentz-TEM is explained.

## RESULTS AND DISCUSSION

### Identification of a multi-ring vortex

**Figure 1.**
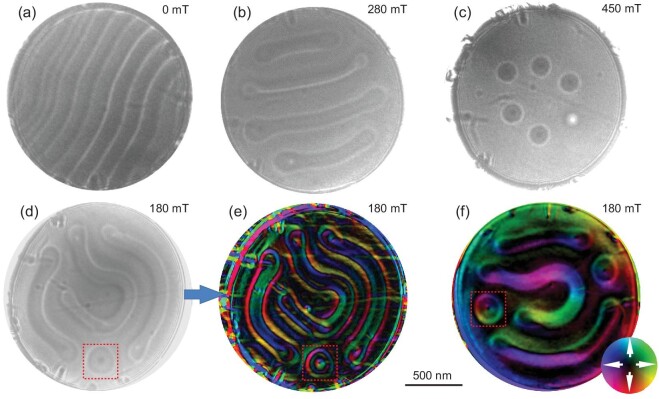
Magnetic field dependence of the spin configurations obtained using Lorentz-TEM at (a) 0 mT, (b) 280 mT and (c) 450 mT. (d) Magnetic configuration obtained by decreasing the field from 450 to 180 mT. (e) The in-plane magnetic configuration from (d) reconstructed using TIE. A magnetic bubble marked by a red dot frame is chosen for the subsequent analysis in Fig. 2. (f) DPC-STEM image of magnetic configuration at ∼180 mT. The spin configurations in (e) obtained by Lorentz-TEM and (f) DPC-STEM are inconsistent because two magnetic imaging modes cannot be directly switched in our TEM setup. The color wheel represents the magnetization direction and amplitude; the dark area suggests the magnetization is out of plane.

**Figure 2.**
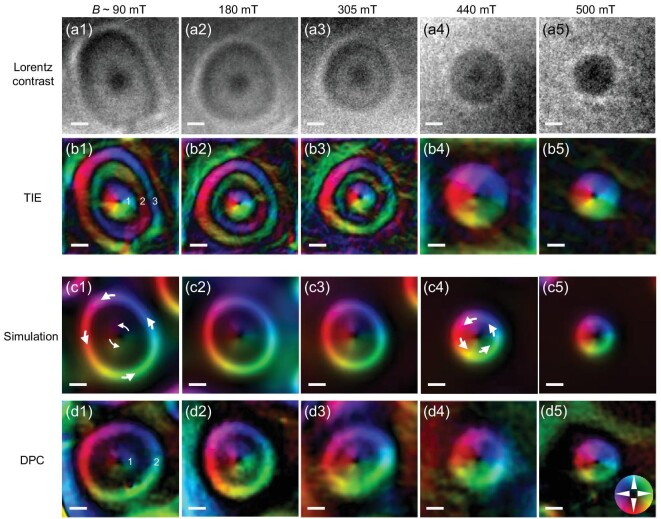
Variations of a magnetic bubble with field. (a1–a5) Intact magnetic contrast under defocused conditions in Lorentz-TEM; the defocus is 500 ${\rm{\mu m}}$. (b1–b5) Magnetic configurations reconstructed by using TIE analysis. At low field (b1, 90 mT; b2, 180 mT; b3, 305 mT), a three-ring magnetic vortex is obtained; the ring number is marked in (b1). At high field (b4, 440 mT; b5, 500 mT), a normal skyrmion is obtained. *B*-dependence of the average in-plane magnetic configurations obtained by simulation (c1–c5) and DPC-STEM (d1–d5). The color wheel in (d5) indicates the direction and strength of the in-plane magnetization. Scale bar: 100 nm.

We first focus on the three-ring vortex in an Fe_3_Sn_2_ uniaxial ferromagnet. An Fe_3_Sn_2_ nanodisk (diameter ∼1550 nm; thickness ∼140 nm) with (001)-oriented out-of-plane direction is chosen for DPC-STEM measurements (Fig. 1f; Fig. S3) and micromagnetic simulations (see the simulation method in the Supplementary Data) [42]. Lorentz-TEM is also performed for comparison. TEM magnetic imaging is discussed in detail in the Supplementary Data [11–13,15,43–49]. Stripe domains are observed at zero field, which transfer into circular domains when a magnetic field is applied out of plane (Fig. 1a–c). However, once the circular domains are formed, they may persist as the field decreases (Fig. 1d). In such a case, the Lorentz-TEM image gives rise to a three-ring vortex at low field (Fig. 1e) that transfers into a normal bubble skyrmion when the field is increased. In Fig. 2a, a field-driven process of one bubble by Lorentz-TEM is shown as an example. At a low field, a black dot in the center is surrounded by outer rings, which is different from a conventional skyrmion image [7,13,19]. The Lorentz contrast of a normal skyrmion is composed of only a black or white circle [5,6,19]. Such distinctness implies complexity in the magnetic objects. When using the TIE method, the reconstructed magnetic configuration is characterized by a series of concentric stripe domains with opposite rotation sense between neighboring magnetic rings (Fig. 2b1–b3), forming a three-ring vortex. At a high field, a normal skyrmion-like image is observed (Fig. 2b4 and b5).

Assuming these nanoscale magnetic objects are arranged in thin nanostructures of uniform magnetization, such complex vortices with multiple rings and field-driven transition cannot be well reproduced in 2D uniaxial ferromagnets. However, we noted that the TEM method can only detect the integral in-plane magnetizations over the depth [5,6,19,45,46]. We noted the *Q* factor of Fe_3_Sn_2_ determined by the ratio of uniaxial magnetic anisotropy (∼54.5 kJm^−3^) to shape anisotropy (∼244 kJm^−3^) is <1. In this case, DDI interaction could lead to the closure of cross-sectional bubble domains, which reveals Néel twisting at the surface and Bloch twisting in the middle [41]. Such Néel twisting at two surfaces of 3D magnetic skyrmion bubbles with contrary chirality has been identified in reciprocal momentum space by a surface-sensitive resonant elastic X-ray scattering in magnetic multilayer films [8,36,37]. Using a nitrogen-vacancy magnetometer, a skyrmion in the surface layer has contrary chirality to intrinsic chiral interaction, which also implies the validity of the proposed 3D magnetic skyrmion bubbles [38]. Furthermore, more complex integral in-plane magnetizations over depth of 3D skyrmion bubbles that are measured using 2D TEM magnetic imaging will be expected and may explain the complex three-ring vortex (Fig. 2b). We thus performed 3D numerical simulations of the Fe_3_Sn_2_ nanodisk, which showed field-driven evolutions of magnetic structures (Fig. S4), similar to those observed in experiments (Fig. 1). The main difference lies in the number of rotationally oriented magnetic rings at a low field. A two-ring vortex of simulated average in-plane magnetizations (Fig. 2c) instead of three-ring vortex in Lorentz-TEM (Fig. 2b) is obtained and characterized by a central weak vortex core and strong circular stripe domain around the edge. Simultaneously, the rotation sense of the outside ring and the central vortex are consistent and anticlockwise here. Such simulated results make sense intuitively because all the interactions in Fe_3_Sn_2_ are achiral. More importantly, such two-ring vortices in simulations (Fig. 2c) are directly visualized by DPC-STEM (Fig. 2d).

The consistency between the simulations and DPC-STEM imaging indicates an artifact in conventional Lorentz-TEM. A filter parameter *q*_0_ is usually used in TIE to increase the signal-to-noise ratio of the reconstructed magnetic structure, avoid divergence and suppress low-frequency disturbance represented by diffraction contrast, thus leading to deviation from the real features [33]. A clear transition from a two-ring magnetic vortex to the multiple-ring vortex with switched circulation is seen as *q*_0_ increases (Fig. S5). Such results imply that the other reported three-ring vortices from TIE analysis of Fresnel images that are not well understood should be re-examined using electronic holography or DPC-STEM to directly acquire the phase shift or phase gradient [26,31].

**Figure 3.**
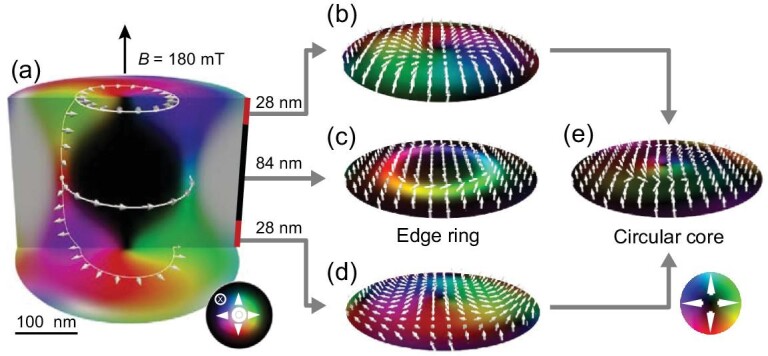
(a) Simulated 3D cross-section spin configurations of a two-ring vortex at 180 mT. (b and d) Average magnetic configuration around the upper and bottom surfaces at 28 nm depth. (e) Average magnetic configuration over the upper and bottom surfaces. (c) Average magnetic configuration around the center at a depth of 84 nm. The color wheels in (a) and (e) represent the in-plane magnetization orientation in (a) and (b)–(e), respectively. The white and darkness in the color wheel in (a) suggest the magnetization is out-of-plane up and down orientations, respectively.

The aforementioned consistency further enables us to analyze the origin of the two-ring vortex. The simulated 3D cross-section spin configuration of a two-ring vortex at a typical field is shown in Fig. 3a. A rugby ball-like 3D structure is obtained, in which hybrid skyrmions along the sample thickness ranged from Néel to Bloch type with increasing depth below the surface, which is attributed to the DDI-induced vortex-like cross-sectional configurations. The sur-face layers host mainly Néel-type skyrmions with radially inward- and outward-pointing spins in the upper and bottom layers, respectively (Fig. 3b and d). The Lorentz-TEM and DPC-STEM only detect the averaged in-plane magnetization, but much of the averaged in-plane magnetization cancels itself out, thus leading to a weak vortex core in the center (Fig. 3e). From the 3D structure, it is readily understood that the outside ring originates from the Bloch-type skyrmions in the middle layers (Fig. 3c), indicating that the two-ring vortex is intrinsically a type-I skyrmion bubble with depth-modulated spin configurations. Interestingly, when the field increased, the size of the outer ring, which comprises contributions from Bloch-type skyrmions in the middle layers, decreases from ∼216 nm at *B* ≈ 90 mT to ∼128 nm at *B* ≈ 450 mT. However, the size of the internal vortex-like core remains constant (∼120 nm). Accordingly, at high field, the internal core and outer ring mix, leading to only one vortex (Fig. S6), which may be responsible for traditional small-size one-ring skyrmion bubbles observed in Fe/Gd films with a comparable *Q* factor as Fe_3_Sn_2_ [41].

Such agreement between the experimental and simulation results verifies the complex 3D structure of the type-I bubble skyrmion, which may give a general understanding of bubble skyrmions in uniaxial ferromagnets with a relatively small *Q* factor [27,41]. We noted that the presented two-ring vortices are distinct from the previously proposed two-ring bubbles in BaFeScMgO [31], which are typically target skyrmions with switched rotations and not attributed to the depth-modulated configurations.

### Identification of an arch-shaped vortex

**Figure 4.**
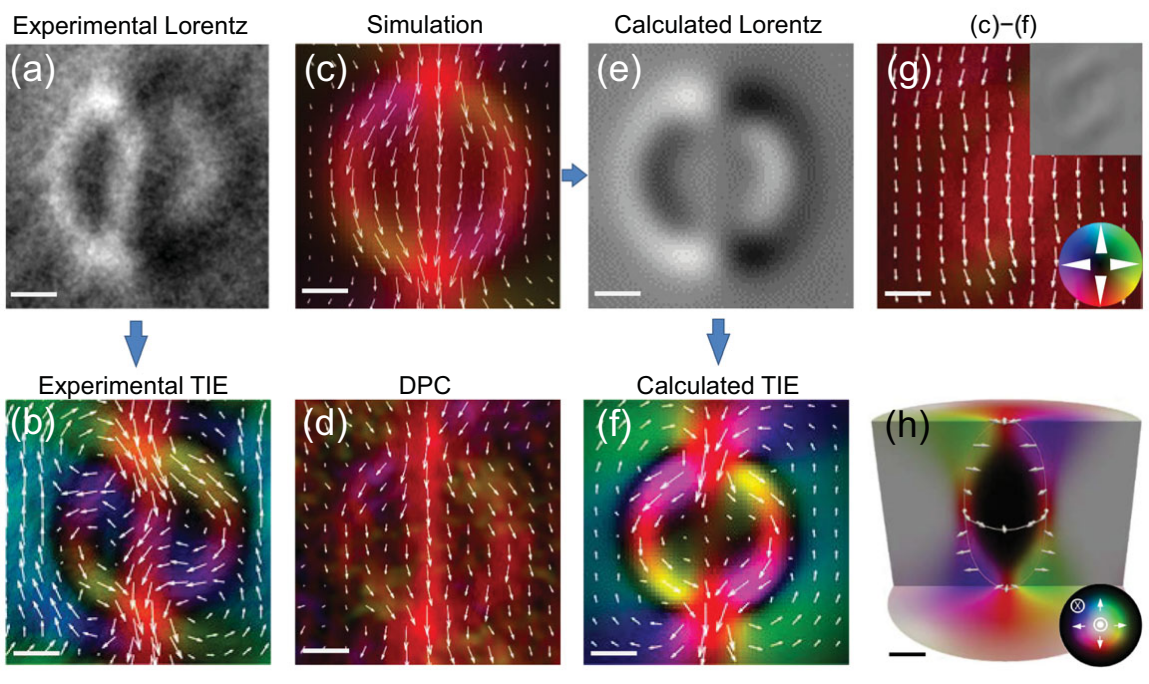
Magnetic configuration of an arch-shaped vortex. (a) The intact magnetic contrast in a defocused Fresnel image in Lorentz-TEM; defocus is 500 ${\rm{\mu m}}$. (b) Magnetic configuration reconstructed using TIE with *q*_0_ = 0. (c) Simulated averaged in-plane magnetization of a type-II magnetic bubble. (d) Representative DPC-STEM images of the magnetic configuration of a type-II bubble. (e and f) Calculated intact Lorentz contrast and the reconstructed magnetic configuration with *q*_0_ = 0 based on the simulations in (c). (g) The difference between (f) and (c). (h) Simulated 3D cross-section spin configuration of the arch-shaped vortex with corresponding averaged in-plane magnetization shown in (c). The color wheels in (g) and (h) represent the in-plane magnetization amplitude and orientation in (b)–(g) and (h), respectively. Scale bar: 50 nm.

Following the procedure outlined previously to investigate the type-I bubble, here we discuss the type-II bubble to clarify the complex arch-shaped vortex [25,31]. According to our experiments, such a vortex can be easily obtained by slightly tilting the external field (Fig. S7). The Lorentz contrast of such a vortex shows Φ-shaped ring with two strong contrasts on the top and bottom (Fig. 4a). A weak line contrast in the center linking the two strong ones is also observed. Using the TIE method, the reconstructed magnetic configuration is characterized by multiple bound pairs of rotating magnetic whirls (Fig. 4b).

The simulated averaged in-plane magnetic configuration (Fig. 4c) shows a Φ-shaped spin whirl with the onion-like characteristic of a type-II bubble [27], which is confirmed using DPC-STEM images (Fig. 4d). Based on the calculated magnetic configuration, the calculated Lorentz contrast (Fig. 4e) is consistent with the experiments (Fig. 4a), thus implying the correctness of the initial Lorentz contrast. However, the magnetic configuration reconstructed by TIE (Fig. 4f) is entirely different from simulations and DPC-STEM images (Fig. 4c and d). Therefore, we believe this magnetic object in Fig. 4b and f is an artificial magnetic configuration created by TIE analysis.

We compared the actual magnetic configuration and artificial magnetic configuration to obtain more insight into this issue. Interestingly, a nearly uniform ferromagnetic background is obtained if we subtract the magnetic configuration in Fig. 4f from that in Fig. 4c. Uniform magnetic configuration can only induce a uniform deflection of the electron beam. However, it cannot provide the Lorentz contrast (inset of Fig. 4g) [50,51]. Therefore, there is no one-to-one correspondence between the Lorentz contrast and a real magnetic configuration. Generally, magnetic objects, differing by only a uniform ferromagnetic background, will exhibit the same Lorentz contrast. In a word, a ferromagnetic magnetization background is easily filtered out from the initial magnetization in the analysis of Lorentz-TEM contrast. We further show that the Φ-shaped spin whirl originates from a rugby ball-like 3D structure ranging from Néel to Bloch type with increasing depth below the surface (Fig. 4h). The outside ring originates from the Bloch-type type-II bubble in the middle layers, and the central line comes from the averaged in-plane magnetization over the upper and bottom surfaces (Fig. S8).

## CONCLUSION

In summary, using DPC-STEM magnetic imaging, we showed that 2D integral magnetizations of 3D type-I and type-II magnetic bubbles can well explain the multi-ring and arch-shaped vortices, respectively. The experimental observations are well reproduced by numerical calculations of real 3D magnetic nanostructures. We further analyzed the intrinsic origin of artifacts of magnetic contrast from Lorentz-TEM. Our results also imply that other unexplained magnetic configurations by TIE should be re-examined using other 2D TEM methods to consider their real 3D magnetic nanostructures [26,31]. In comparison to surficial magnetic configurations of 3D magnetic structures revealed by surface-sensitive methods [36–38], we provide a proof of the 3D magnetic bubbles in nanostructures from the view of 2D integral magnetizations. Given that the two types of  bubbles are nanoscale magnetic objects, the next step is to study the dynamics induced by current to build a purely bubble-based spintronic device [28].

## METHODS

We prepared bulk Fe_3_Sn_2_ crystals by chemical vapor transport and fabricated the Fe_3_Sn_2_ nanodisk using a focused ion beam and scanning electron microscopy dual-beam system (Helios NanoLab 600i, FEI). The magnetic imaging of the Fe_3_Sn_2_ nanodisk was performed on a TEM (Talos F200X, FEI) operated at 200 kV. Micromagnetic simulations were performed using a GPU-accelerated program: MuMax3. For details about the methods, refer to the Supplementary Data.

## Supplementary Material

nwaa200_Supplemental_File
