# Supplementary material for: Two-dimensional characterization of three-dimensional magnetic bubbles in Fe3Sn2 nanostructures
Source: Natl Sci Rev. 2020 Aug 28;8(6):nwaa200. doi: 10.1093/nsr/nwaa200 (PMC8288175; doi:10.1093/nsr/nwaa200)
Supplement: nwaa200_Supplemental_File [file nwaa200_supplemental_file.zip › 3D_bubble_supplemnetal.docx]

**Supporting Information:**

**Two-dimensional characterization of three-dimensional nanostructures of magnetic bubbles in Fe3Sn2**

Jin Tang1, Yaodong Wu1,2, Lingyao Kong3, Weiwei Wang4, Yutao Chen1, Yihao Wang1, Y. Soh5, Yimin Xiong1, Mingliang Tian1,3, and Haifeng Du1,4*

1Anhui Province Key Laboratory of Condensed Matter Physics at Extreme Conditions, High Magnetic Field Laboratory of the Chinese Academy of Sciences, and University of Science and Technology of China, Hefei, 230031, China

2Universities Joint Key Laboratory of Photoelectric Detection Science and Technology in Anhui Province, Hefei Normal University, Hefei, 230601, China

3Schoolof Physics and Materials Science, Anhui University, Hefei, 230601, China

4Institutes of Physical Science and Information Technology, Anhui University, Hefei, 230601, China

5Paul Scherrer Institute, 5232, Villigen, Switzerland

*Corresponding author: duhf@hmfl.ac.cn

**Methods**

***Bulk sample preparations:*** Single Fe3Sn2 crystals were grown by chemical vapor transport with stoichiometric iron (Alfa Aesar, >99.9%) and tin (Alfa Aesar, >99.9%). The sintered Fe3Sn2 was obtained by heating the mixture at 800°C for 7 days, followed by thorough grinding. It was then sealed with I2 in a quartz tube under vacuum and kept in a temperature gradient of 720°C to 650°C for 2 weeks. The crystal quality and structure group were both verified using Cu *Kα* radiation (TTR3 diffractometer, Rigaku).

***Fabrication of the Fe3Sn2 nanostructures:*** The thin Fe3Sn2 nanodisc used for TEM imaging had a diameter of 1550 nm and thickness of ca. 140 nm. It was fabricated by a lift-out process using a focused ion beam and scanning electron microscopy dual beam system (Helios NanoLab 600i, FEI) combined with a gas injection system and micromanipulator (OmniProbe 200+, Oxford). The nanodisc fabrication process was previously reported in detail [1,2].

***TEM measurements:*** High-resolution crystal structure and magnetic imaging were conducted in a TEM (Talos F200X, FEI) operated at 200 kV. Both Lorentz transmission electronic microscopy (Lorentz-TEM) and differential phase contrast (DPC) scanning TEM modes were used to obtain magnetic imaging, which can be qualitatively understood in terms of the Lorentz force expressed by **F**=–*e*(**v**× **B**) acting on the electrons transmitted through a magnetic foil, where *e*, **v**, and **B**are the electric charge, the velocity of electrons, and magnetic field, respectively. Because the motion of the transmitted electrons is affected only by the magnetic induction perpendicular to the electron projection direction (out-of-plane), only the in-plane field (*Bxy*) can be detected using TEM in principle. The deﬂection angle *βL* of an electron induced by a magnetic field can be expressed as *βL =* *Bxye* [3], where is the wavelength of an elecrtron and is Planck’s constant. Alternatively, based on the Aharonov–Bohm eﬀect, the eﬀect of the magnetic induction can be described as a phase shift  in quantum mechanics [4]. The phase gradient is derived to be proportional to the deflection angle and expressed as, [3]. The magnetic field *B* that is proportional to the deflection angle *L* can be obtained from the phase gradient . The magnetic imaging using TEM is thus able to be realized by some special modes to acquire the phase shift . For varying field magnetic imaging, the objective lens of the microscope was turned off and adjusted to provide an out-of-plane magnetic field within a field ranging from −1700 to 1700 mT, as calibrated by a standard Hall probe. Traditional Lorentz-TEM acquires the phase shift from the Fresnel images via a transport of intensity equation (TIE) process.

The DPC microscope was operated at low magnification in scanning TEM (STEM) mode using a split quadrant detector (Figure S2). The probe convergence and detection angles for the DPC-STEM measurements were set to 7 and 1 mrad, respectively; the corresponding probe size is ~3.6 nm. The beam deflection angle *βL* of the focused electron beam that is proportional to the phase gradient is directly obtained from the intensity in each quadrant, named A, B, C, and D (Figure S2), of the segmented detector [3]. The orthogonal phase gradients along the *x* axis () and the *y* axis (), are obtained by subtracting the signals of two orthogonal detectors, that is, differences and , respectively. The phase gradients and are proportional to the magnetic field –*By* and –*Bx*, respectively, by considering the phase shift induced by magnetic field according to Equation (12). The phase shift is linear to an integral DPC (iDPC) image [5]. The strengths of the in-plane field (**Bxy**) were thus obtained through quadratic summation of the image differences (). The field orientation was determined by implementing the arctan of [(A–C)/(B–D)]. A typical analysis of DPC-STEM is shown in Figure S3. Note that our DPC-STEM setup cannot distinguish the phase gradient induced by electric and magnetic fields. The electric field reveals important information near a defect or the sample edge in our experiment and is thus ignored during the analysis of magnetic contrast.

***Micromagnetic simulations* [6]**: The micromagnetic simulations were performed using a GPU-accelerated program: Mumax3. The total free energy terms are written as:

, (1)

where , , , and . Here is the normalized continuous vector field representing the magnetization , and is a unit vector of uniaxial magnetic anisotropy. *A*, *Ku*,and *Ms* are the exchange interaction, uniaxial magnetic anisotropy constant, and saturation magnetization, respectively. **B**d is the demagnetizing field. The saturation magnetization *Ms* of Fe3Sn2 is obtained from the magnetization of bulk Fe3Sn2 at a high field (7 T) at 300 K. The uniaxial magnetic anisotropy constant was determined from the saturation field (*Hk ≈*171mT) of the bulk Fe3Sn2 along the *ab* plane at 300 K by a function *Ku* = 1/2*HkMs*. The exchange interaction constant *A* was adjusted to fit the average stripe domain width (ca. 175 nm), as shown in Figures 1(a) and S2a. Here we set the material parameters *Ms =* 622.7 kA m−1, *A* = 8.25 pJ m−1, and *Ku* = 54.5 kJ m−3, which are all typical parameters of the Fe3Sn2 uniaxial ferromagnet at room temperature. The cell size is set as 4 4 4 nm3. The equilibrium spin configuration was obtained by using a conjugate gradient method.

***Simulation of the Lorentz-TEM image*** [7,8]: We obtained the configurations of the magnetic phase shift images based on the Aharonov–Bohm equation. The magnetic phase shift for an electron traversing the *z* axis is [9]:

, (2)

where,  is the magnetic vector potential. Magnetic vector potential has the contributions of demagnetization field**Hd** with an expression as . For a magnetic object with magnetization , the magnetic vector potential is derived from the classic electrodynamics and expressed as [9]:

(3)

Combing the Euqations (2) and (3), we can obtained the magnetic phase shift and expressed as follows [8,9]:

, (4)

where and are the in-plane magnetization components that have been averaged over the *z* axis. They are expressed by: , and , respectively. We performed an integration over the thickness of the material along the line defined as *x* = *x*’ or *y* = *y*’. When the electrons have passed through the structure, thus acquiring a magnetic phase, they reach the back focal plane of the objective lens. Here, for simplicity, we only considered the contribution of the magnetic phase to the Lorentz-TEM image. The electron disturbance can be computed by performing a Fourier transform on the wave function of the transmitted electron beam:

. (5)

The electron wave function is modified to using a transfer function:

, (6)

where , , and are the relativistic wavelength of the electrons, aberration coefficient of the objective lens, and defocus distance, respectively, and *k* is expressed by . We assumed the pupil function was constant for all reciprocal space. In actual experiments, the Lorentz-TEM resolution is affected by the spread of the electron source and spatial coherence, which must be considered. We used an envelope function to describe the spread of the source, which is expressed as a Gaussian distribution:

. (7)

Here, is the beam divergence angle. The Lorentz-TEM intensity at the screen can be finally obtained as:

. (8)

We set = 0.0025 nm, thus corresponding to 200 keV electrons, and *Cs* = 0 nm during the simulations. The beam divergence is set as 0 mrad if it is not specified.

***Spin configuration obtained from magnetic TIE analysis*** **[10-12]**: Three Lorenz-TEM intensity contrast images at different defocus values (de-, in-, and over-focus) were analyzed by using the TIE analysis, and the phase shift can be obtained based on:

. (9)

We further used a Fourier transition and replaced the inverse Laplacian by:

. (10)

Here, *q*(*x, y*) is the spatial frequency in the image plane. Typically, a filter parameter *q*0 is used to avoid divergence and suppress low-frequency noise represented by diffraction contrast, that is, to increase the signal-to-noise ratio of the obtained magnetic structure. Equation (8) must be replaced by:

. (11)

Finally, we reconstructed the in-plane magnetic field from the following expression:

, (12)

where, and *t* are the reduced Planck constant and material thickness, respectively, and is the unit vector parallel to the beam direction. The influence of the filter parameter *q0* on the retrieved magnetic structure is shown in Figure S5.

***Analyzing spin configurations in the micromagnetic simulations, simulated Lorentz-TEM images,* *and TIE analysis*:** We first obtained an initial equilibrium spin configuration in micromagnetic simulations using a conjugate gradient method. We then obtained the corresponding phase shift based on Equation (4). We further obtained the simulated Lorentz-TEM images under de-, in-, and over-focus conditions from the phase shift based on Equation (8). The corresponding phase shift can be further inversely obtained using the typical TIE analysis from the simulated Lorentz-TEM contrast images based on Equations (9)–(11). Finally, the magnetic field was reconstructed from the TIE analysis based on Equation (12).

**TEM MAGNETIC IMAGING**

The basic principle of TEM magnetic imaging can be understood classically in terms of the deflection of the electron beams induced by the magnetic field **B**, as described in the Lorentz force law. Alternatively, a phase shift of an electronic wave can be induced by the magnetic field based on the Aharonov–Bohm eﬀect in quantum mechanics [4]. It is thus derived that the phase shift gradient is proportional to the deflection [3]. Therefore, TEM magnetic imaging can be realized by some special modes to acquire a phase shift. Off-axis electronic holography, Lorentz-TEM (also called as in-line electron holography), and DPC-STEM are three main methods of TEM magnetic imaging [13]. Off-axis electronic holography directly acquires the phase shift from changes in the spacing of the interference fringes of the electron wave results from a biprism passing through a vacuum and magnetic field [13,14]. However, the sample size for electronic holography is limited to the effective width of the interference fringes for high-resolution magnetic imaging (typically ~1 for our TEM setting). Therefore, here we mainly use Lorentz-TEM and DPC for magnetic imaging of a 1550-nm Fe3Sn2 nanodisc.

The most commonly used technique in TEM magnetic imaging is the Fresnel method that obtains the phase shift using TIE analysis [10-13]. The Fresnel magnetic contrast originates from the superposition of two electron beams passing through two magnetic domains at out-of-focus conditions (see supplementary Figure S2a) [13]. As a result, Fresnel imaging in Lorentz-TEM is limited to domain walls, and uniform in-plane ferromagnetic magnetization leads to uniform Lorentz contrasts. Unlike the Lorentz-TEM, DPC-STEM relies on a quadrant-segmented detector that is directly used to obtain the deflection of a focused electron beam (Figure S2b). Accordingly, this technique directly detects the phase gradient along two orthogonal directions from two orthogonal subtracted detector signals (Figure S3). Furthermore, DPC-STEM is operated at an in-focus case, which avoids strong Fresnel fringes from defects or sample edges compared with Lorentz-TEM. Therefore, DPC-STEM has the advantages of in-focus STEM imaging and direct access to the phase gradient enable to visual domains and fine magnetic structures of skyrmions [15-17].

Other TEM imaging methods, such as electron tomography and ptychography, have been shown to have ultrahigh spatial resolution and even the ability to visualize 3D structures [14,18], but their applications on magnetic imaging are rare because of some unexplored issues. For example, electron tomography reconstructs 3D structures from a series of images obtained from a continuously rotating sample; such a process will introduce magnetic structure variations for a given out-of-plane field that renders magnetic structure reconstruction impossible. In the present case, electron tomography is expected to obtain zero-field 3D magnetic structures using field-free operations. Imaging 3D magnetic structures using electron tomography is expected to have wide applications in the developing rotatory field along with sample rotation.

For varying field magnetic imaging, the objective lens current was adjusted to provide an out-of-plane magnetic field, as calibrated by a standard Hall probe, in the Lorentz-TEM and DPC-STEM modes. DPC-STEM and Lorentz-TEM cannot be directly switched because the operation of switching magnetic imaging modes from Lorentz-TEM to DPC-STEM must be conducted under a demagnetizing field process from ~1700 mT in our present TEM setup.
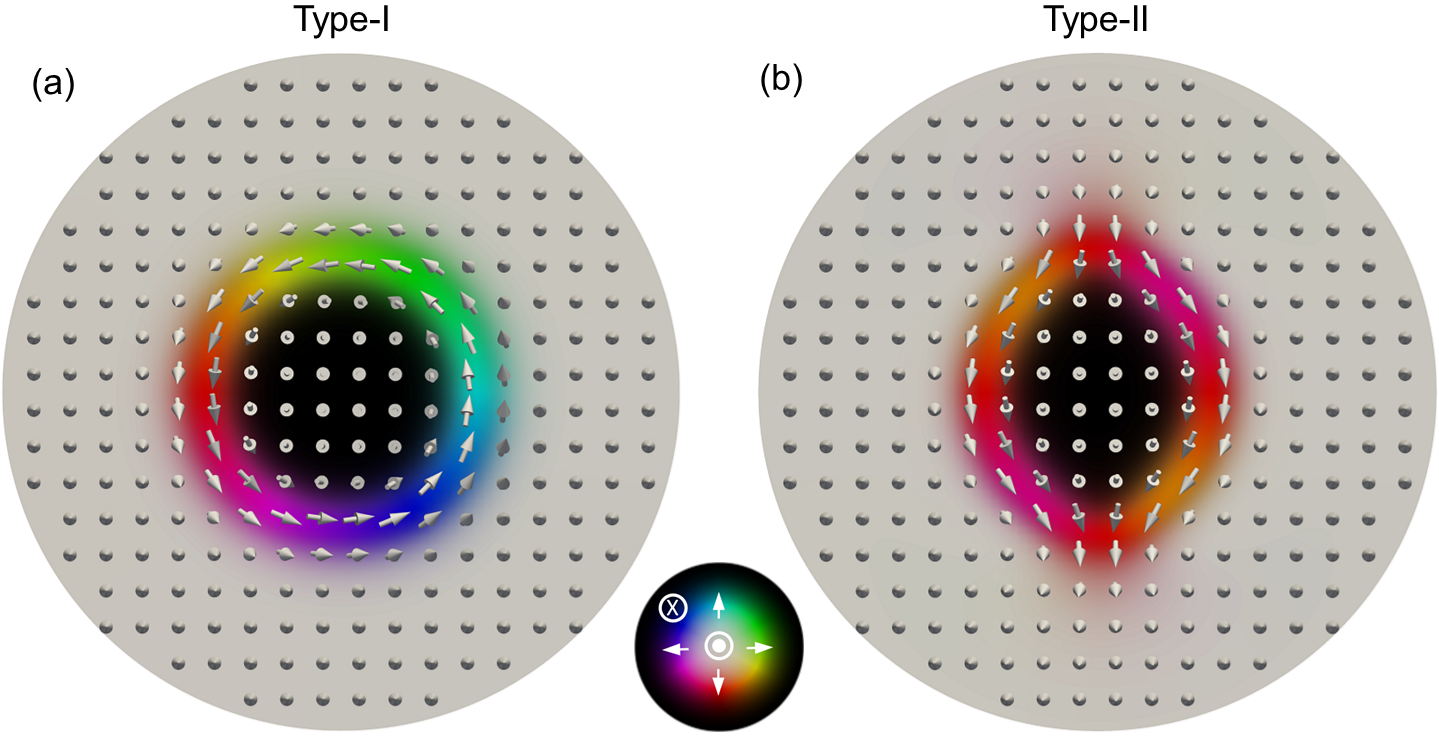
Figure S1. Representative schematic magnetic configurations for (a) a type-I bubble and (b) a type-II bubble. The color wheel indicates the direction of magnetization at each point; the white and darkness suggest magnetization point out-of-plane up and down orientation, respectively.


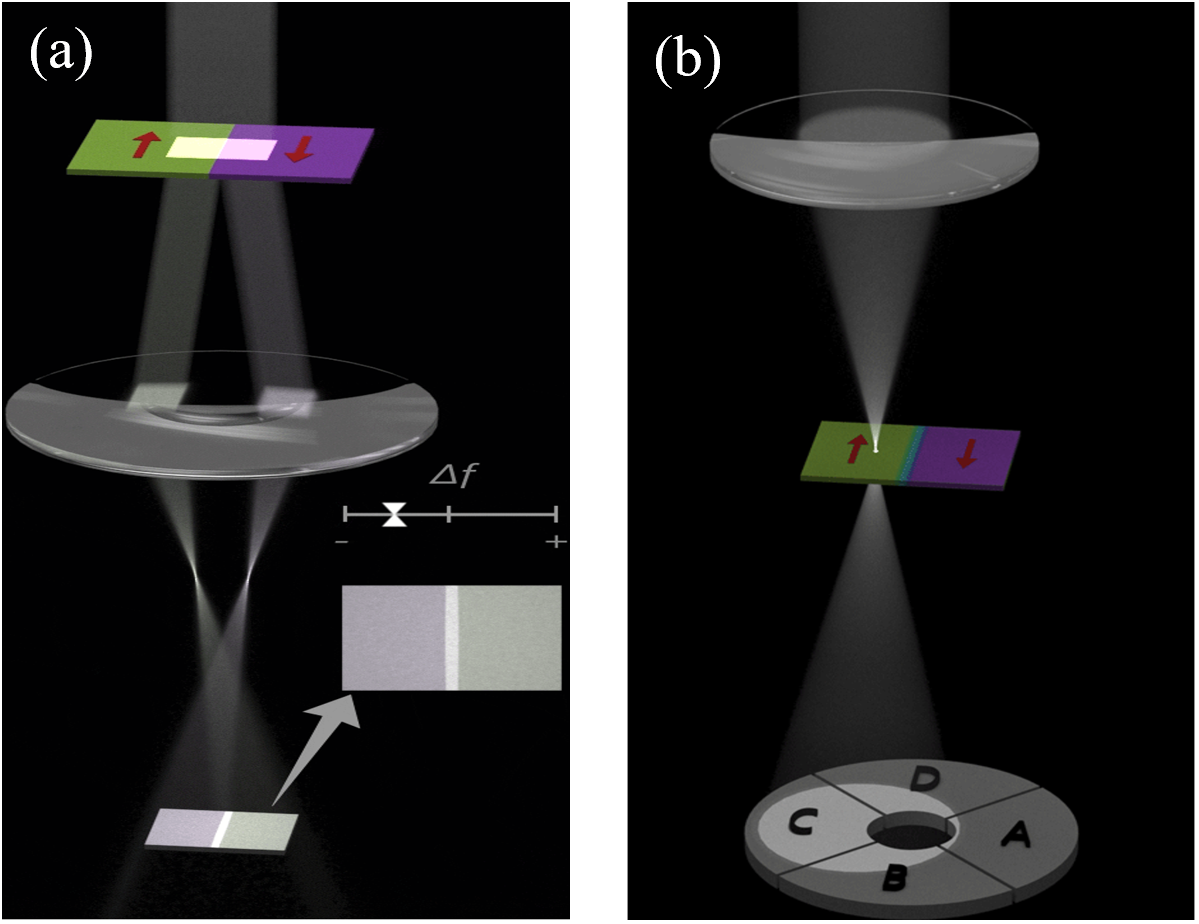


**Figure S2.** (a) Schematic ray diagram in a Fresnel image of a ferromagnetic domain specimen containing one 180° domain wall. A bright contrast around the domain wall forms by the superposition of two deflected electron beams. The appearance of dark or bright contrast depends on the out-of-focus conditions tuned by the focus distance . (b) Schematic ray diagram in a DPC-STEM image. A beam of electrons is focused on a probe and scanned across the specimen. The beam deflected by the Lorentz force is collected using a detector with four segments. By considering the difference in the signals across the opposite quadrants, the direction and magnitude of the field can be directly deduced in real space, and the complete in-plane magnetic configurations can be mapped irrespective of the domain or domain wall.


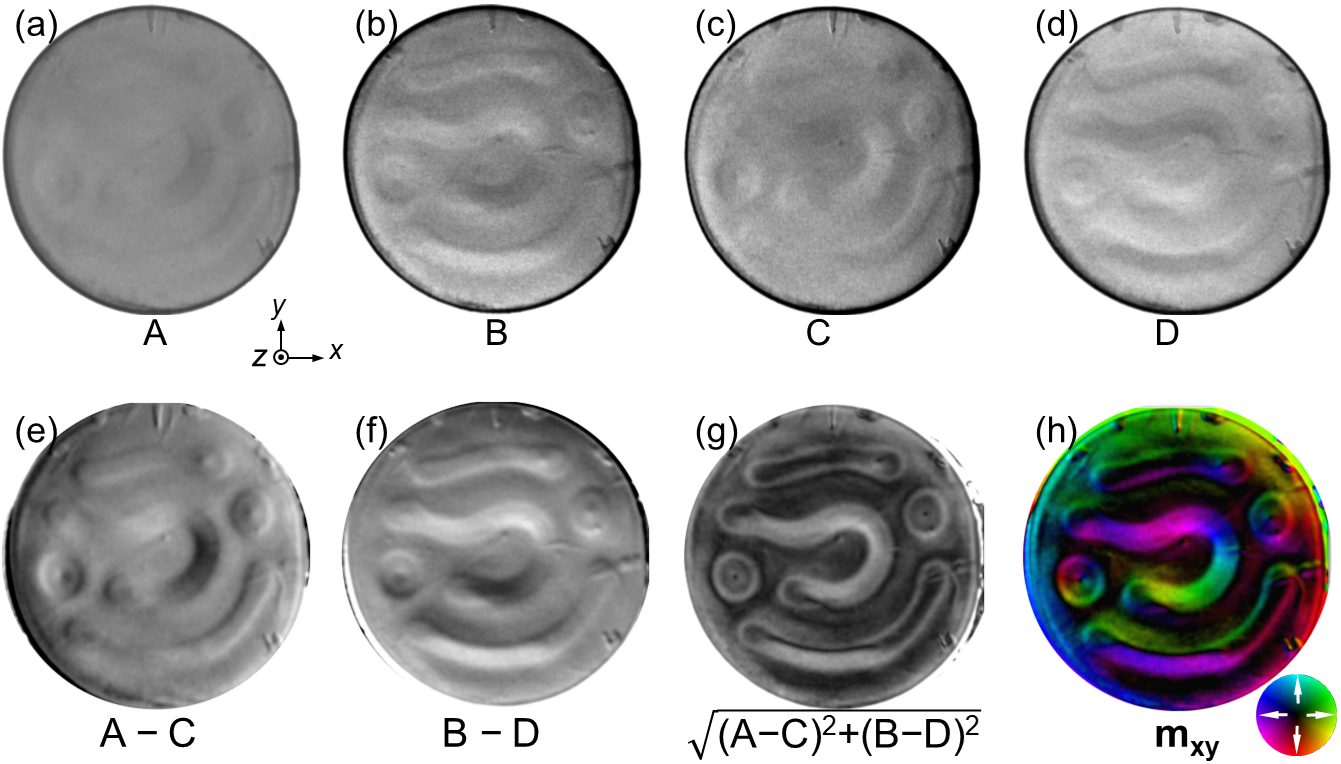


**Figure S3.** Analysis procedure for determining the magnetic structure in a 1550 nm Fe3Sn2 disc by using DPC-STEM.(a)–(d) DPC component images from the four segments of the detectors A, B, C, and D, respectively. (e) DPC component obtained by subtracting C from A () to detect the phase gradient along the *x* axis , which is proportional to the field component along the *y* axis (**By**)*.* (f) DPC component obtained by subtracting D from B () to detect the phase gradient along the *y* axis , which is proportional to the field component along the *x* axis (**Bx**)*.* (g) Total in-plane field strength (|**Bxy**|) obtained from . (h) Color mapping of in-plane field magnetization with direction and amplitude depicted based on the color wheel shown in the inset. The field direction is obtained by arctan[(A–C)/(B–D)]. The in-plane magnetization inside the magnetic sample (**mxy**) is proportional to the in-plane field (**Bxy**)*.*


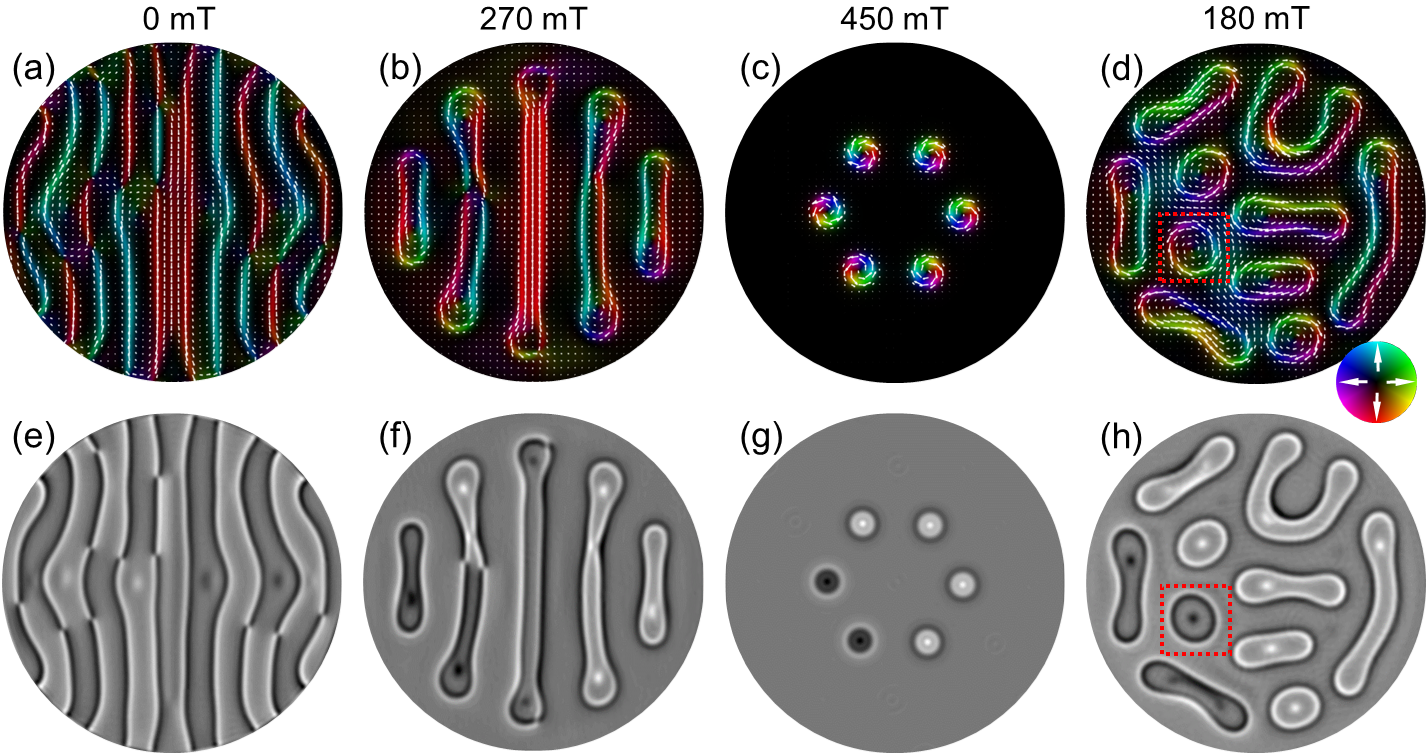


**Figure S4.** Simulated evolution of the magnetic structure in the 1550 nm ferromagnetic disc as a function of the external magnetic field.(a)–(c) Field-driven transition from initial stripe domains at the zero field to a bubble cluster at 450 mT. (d) Magnetic structure obtained by decreasing the magnetic field from 450 mT to 180 mT. (e)–(h) Corresponding simulated Lorentz contrast images at 0, 270, 450, and 180 mT taken under defocused conditions; defocus is 500 .


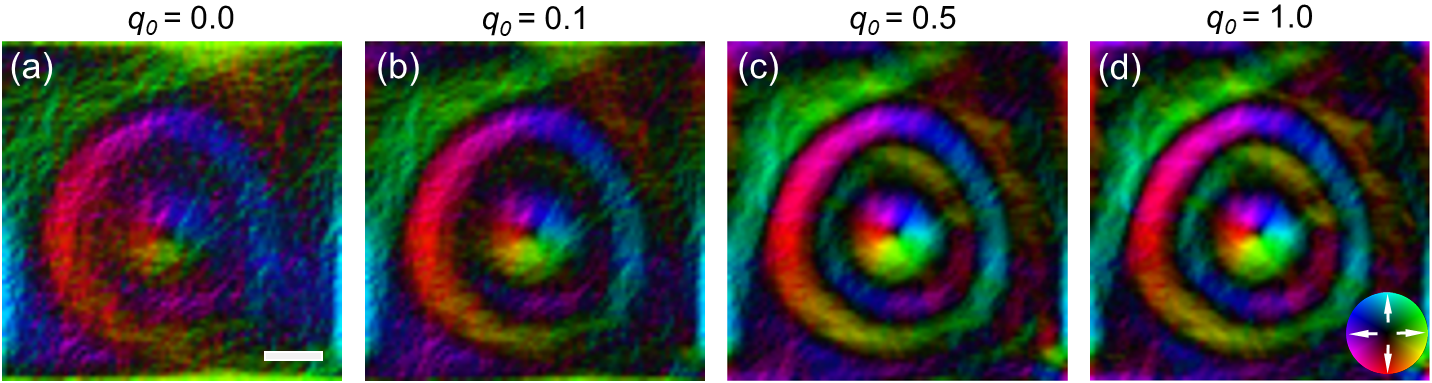


**Figure S5.** Reconstructed type-I bubble magnetic structure by TIE analysis with the filter parameter *q0* = 0.0 (a), *q0* = 0.1 (b), *q0* = 0.5 (c), and *q0* = 1.0 (d). The parameter *q0* significantly increases the signal-to-noise ratio of the retrieved magnetic structure. However, the magnetic structure transformed from a two-ring vortex into a three-ring vortex as *q0* increased. The scale bar is 100 nm.


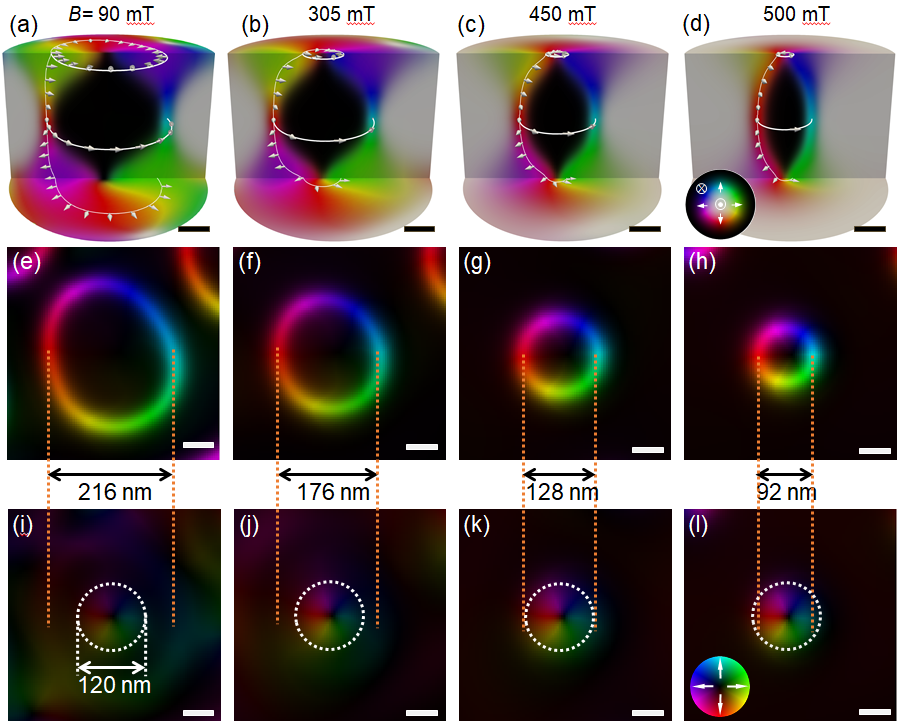
**Figure S6.** Field-driven evolution of a type-I bubble structure, as obtained by numerical simulation. Three-dimensional magnetic structures at (a) *B* = 90, (b) 305, (c) 450, and (d) 500 mT. Thein-plane magnetization mapping with amplitude and orientation in (a)–(d) are plotted according to the color wheel shown in the inset of (d). The averaged in-plane magnetization with contributions from the layers near the internal middle layers (84 nm) at (e) 90, (f) 305, (g) 450, and (h) 500 mT. The averaged in-plane magnetization with contributions from the layers near the surface (56 nm) at (i) 90, (j) 305, (k) 450, and (l) 500 mT. The in-plane magnetization mapping with amplitude and orientation in (d)–(l) are plotted according to the color wheel shown in the inset of (l). All scale bars are 50 nm. The diameters of the dotted circles in (i)–(l) are 120 nm.


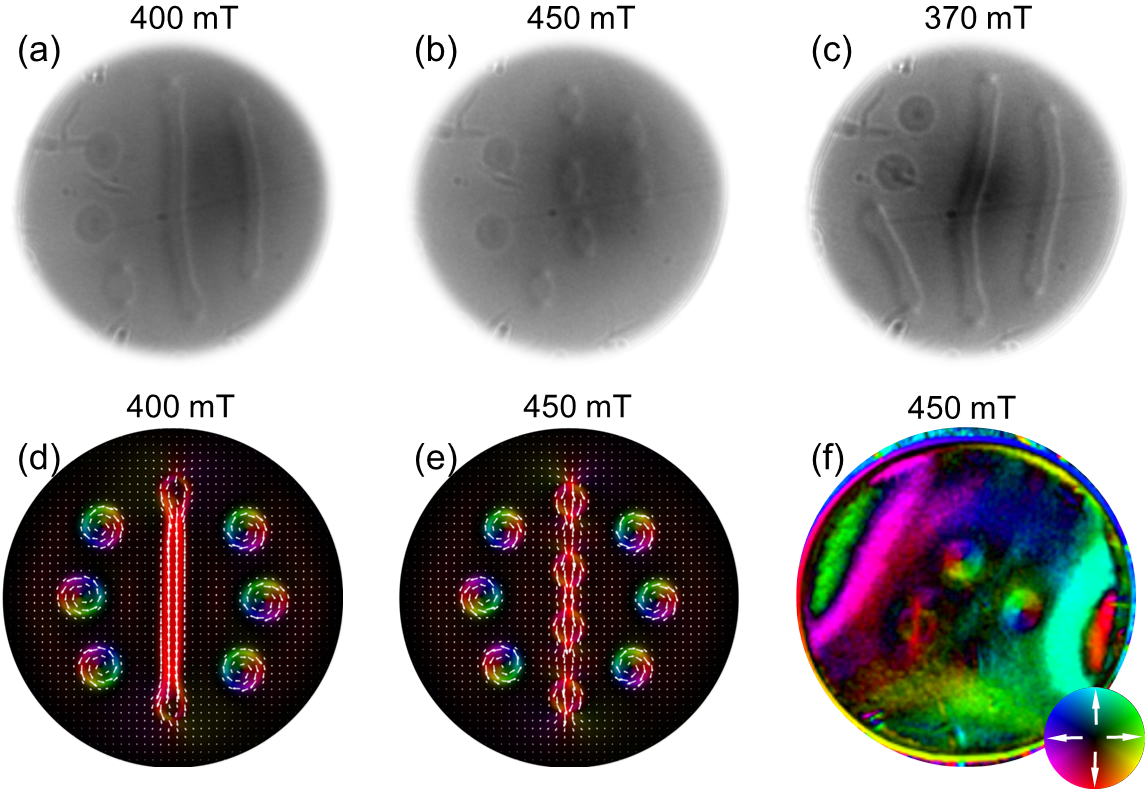
**Figure S7.** Transitions between the stripe domain and type-II bubble at a tilted magnetic field; angle is ca. 2.0 deg in the 1550 nm nanodisc. (a) Stripe domains with mixed bubbles at *B* ≈ 400 mT by Lorentz-TEM. (b) Mixed type-I and type-II bubbles at *B* ≈ 450 mT by Lorentz-TEM. (c) Transition from a type-II bubble to a stripe domain at *B* ≈ 370 mT by Lorentz-TEM. (d)–(e) Simulated transition from a stripe domain at *B* = 400 mT to a type-II bubble at *B* = 450 mT. (f) Magnetic bubbles obtained by DPC-STEM at *B* = 450 mT.


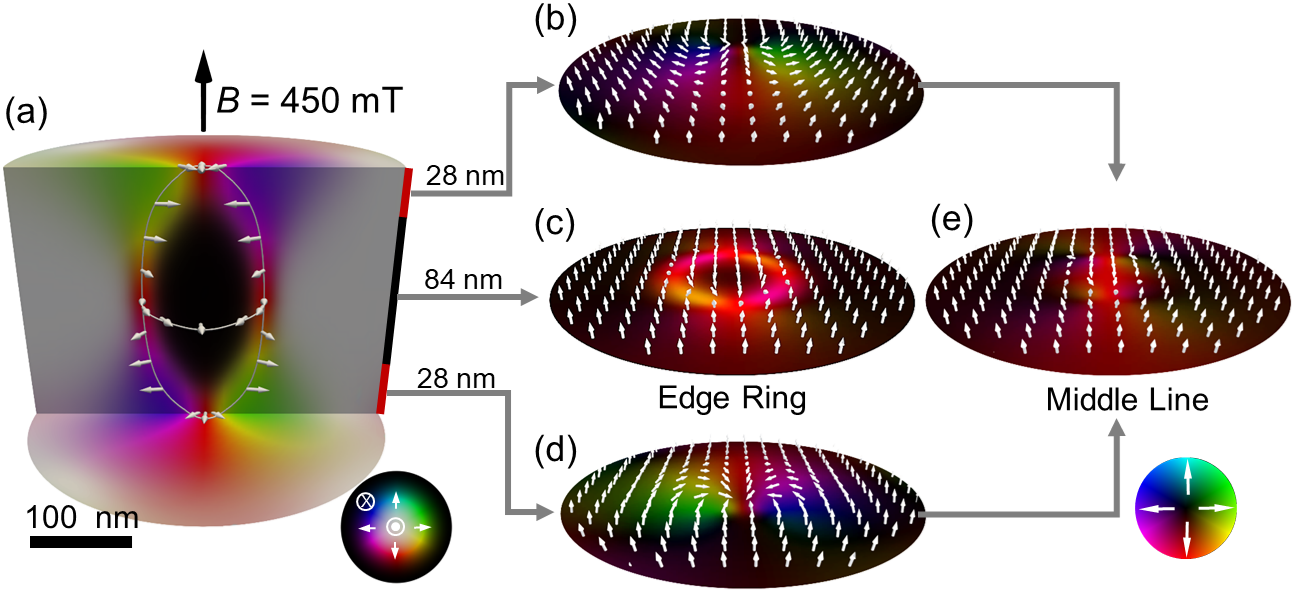


**Figure S8.** Three-dimensional depth-modulated magnetic structure of a type-II bubble.(a) Three-dimensional and cross-sectional spin configurations of a type-II bubble at 450 mT. Inset shows the color wheel representing the in-plane magnetization orientation and amplitude. (b) The averaged magnetization mapping in the top layers with 28-nm depth. (c) The averaged magnetization mapping in the middle layers with 84-nm depth revealing the edge ring. (d) The averaged magnetization mapping in the bottom layers with 28-nm depth. (f) Superposition of the top layers (b) and the bottom layers (d) revealing the middle line. The color mapping represents the in-plane orientation and amplitude shown in (b)–(e) are plotted according to the right color wheel.

**REFERENCES**

1. Zheng F, Li H and Wang S *et al.* Direct Imaging of a Zero-Field Target Skyrmion and Its Polarity Switch in a Chiral Magnetic Nanodisk. *Phys Rev Lett* 2017; **119**: 197205.

2. Zhao X, Jin C and Wang C *et al.* Direct imaging of magnetic field-driven transitions of skyrmion cluster states in FeGe nanodisks. *Proc Natl Acad Sci U S A* 2016; **113**: 4918.

3. Krajnak M, McGrouther D and Maneuski D *et al.* Pixelated detectors and improved efficiency for magnetic imaging in STEM differential phase contrast. *Ultramicroscopy* 2016; **165**: 42-50.

4. Aharonov Y and Bohm D. Significance of Electromagnetic Potentials in the Quantum Theory. *Phys Rev* 1959; **115**: 485-491.

5. Lazic I, Bosch EGT and Lazar S. Phase contrast STEM for thin samples: Integrated differential phase contrast. *Ultramicroscopy* 2016; **160**: 265-280.

6. Vansteenkiste A, Leliaert J and Dvornik M *et al.* The design and verification of MuMax3. *AIP Adv* 2014; **4**: 107133.

7. Walton SK, Zeissler K and Branford WR et al. MALTS: A tool to simulate Lorentz Transmission Electron Microscopy from micromagnetic simulations. *arXiv* 2012; **1207**: 2310.

8. Nayak AK, Kumar V and Ma T *et al.* Magnetic antiskyrmions above room temperature in tetragonal Heusler materials. *Nature* 2017; **548**: 561-566.

9. Zheng F, Rybakov FN and Borisov AB *et al.* Experimental observation of chiral magnetic bobbers in B20-type FeGe. *Nat Nanotechnol* 2018; **13**: 451-455.

10. Volkov VV and Zhu Y. Phase imaging and nanoscale currents in phase objects imaged with fast electrons. *Phys Rev Lett* 2003; **91**: 043904.

11. Paganin D and Nugent KA. Noninterferometric Phase Imaging with Partially Coherent Light. *Phys Rev Lett* 1998; **80**: 2586-2589.

12. Jin C, Li ZA and Kovacs A *et al.* Control of morphology and formation of highly geometrically confined magnetic skyrmions. *Nat Commun* 2017; **8**: 15569.

13. Tang J, Kong L and Wang W *et al.* Lorentz transmission electron microscopy for magnetic skyrmions imaging. *Chin Phys B* 2019; **28**: 087503.

14. Midgley PA and Dunin-Borkowski RE. Electron tomography and holography in materials science. *Nat Mater* 2009; **8**: 271-280.

15. Shibata N, Findlay SD and Matsumoto T *et al.* Direct Visualization of Local Electromagnetic Field Structures by Scanning Transmission Electron Microscopy. *Acc Chem Res* 2017; **50**: 1502-1512.

16. McGrouther D, Lamb RJ and Krajnak M *et al.* Internal structure of hexagonal skyrmion lattices in cubic helimagnets. *New J Phys* 2016; **18**: 095004.

17. Matsumoto T, So Y-G and Kohno Y *et al.* Direct observation of Σ7 domain boundary core structure in magnetic skyrmion lattice. *Sci Adv* 2016; **2**: e1501280.

18. Gao S, Wang P and Zhang F *et al.* Electron ptychographic microscopy for three-dimensional imaging. *Nat Commun* 2017; **8**: 163.
